# Melanism evolution in the cat family is influenced by intraspecific communication under low visibility

**DOI:** 10.1371/journal.pone.0226136

**Published:** 2019-12-18

**Authors:** Maurício Eduardo Graipel, Juliano André Bogoni, Eduardo Luís Hettwer Giehl, Felipe O. Cerezer, Nilton Carlos Cáceres, Eduardo Eizirik

**Affiliations:** 1 Departamento de Ecologia e Zoologia, Centro de Ciências Biológicas, Universidade Federal de Santa Catarina, Florianópolis, Santa Catarina, Brazil; 2 Laboratório de Ecologia, Manejo e Conservação de Fauna Silvestre (LEMaC), Escola Superior de Agricultura “Luiz de Queiroz”, Universidade de São Paulo, Piracicaba, São Paulo, Brazil; 3 Programa de Pós-Graduação em Ecologia, Centro de Ciências Biológicas, Universidade Federal de Santa Catarina, Florianópolis, Santa Catarina, Brazil; 4 Programa de Pós-Graduação em Biodiversidade Animal, CCNE, Universidade Federal de Santa Maria, Santa Maria, Rio Grande do Sul, Brazil; 5 Departamento de Ecologia e Evolução, CCNE, Universidade Federal de Santa Maria, Santa Maria, Rio Grande do Sul, Brazil; 6 Escola de Ciências da Saúde e da Vida, Pontifícia Universidade Católica do Rio Grande do Sul, Porto Alegre, Rio Grande do Sul, Brazil; University of Georgia, UNITED STATES

## Abstract

Melanism in the cat family has been associated with functions including camouflage, thermoregulation and parasite resistance. Here we investigate a new hypothesis proposing that the evolution of melanism in cats has additionally been influenced by communication functions of body markings. To evaluate this hypothesis, we assembled a species-level data set of morphological (body marks: white marks on the backs of ears) and ecological (circadian activity: arrhythmic/nocturnal, and environmental preference: open/closed) characteristics that could be associated with communication via body markings, and combined these data with a dated molecular phylogeny. Next, we tested the association between melanism and communication, first by relating species’ body marks with their ecological conditions, using a Bayesian implementation of the threshold model. Second, to explore the evolution of characteristics potentially influencing melanism in cat species, we modeled their evolution relative to melanism using models of coordinated *vs*. independent character changes. Our results suggest that white marks are associated with intraspecific communication between individuals that have non-melanistic phenotypes, as well as towards melanistic individuals (without white marks). The absence of white marks in a melanistic individual tends to be a limiting condition for intraspecific visual communication at night, resulting in an evolutionary dilemma for these species, i.e. to be almost invisible at night, but not to communicate visually. The comparative analysis of several evolutionary models indicated more support for the evolution of melanism being coordinated with the evolution of arrhythmic activity and white marks on the backs of ears.

## Introduction

Melanism in the cat family has usually been proposed to be associated with biological factors such as camouflage, thermoregulation, parasites, and sexual selection [1, 2, 3, 4] and ecological factors such as circadian activity and habitat use [4, 5, 6]. More broadly, camouflage from predators, *e*.*g*. in lizards [7], rock pocket mice [8] and tree squirrels [9], and thermal advantage, *e*.*g*. in butterflies, ladybirds, snails and snakes [10, 11] have been proposed as the two major functions of melanism [9]. These studies indicate that melanism is a pervasive phenomenon, and can be used as a model to explore the forces and dynamics driving evolutionary change [2, 12]. Still, the key proximate factors driving the evolution of melanism in cat species remain incompletely understood.

**Fig 1:**
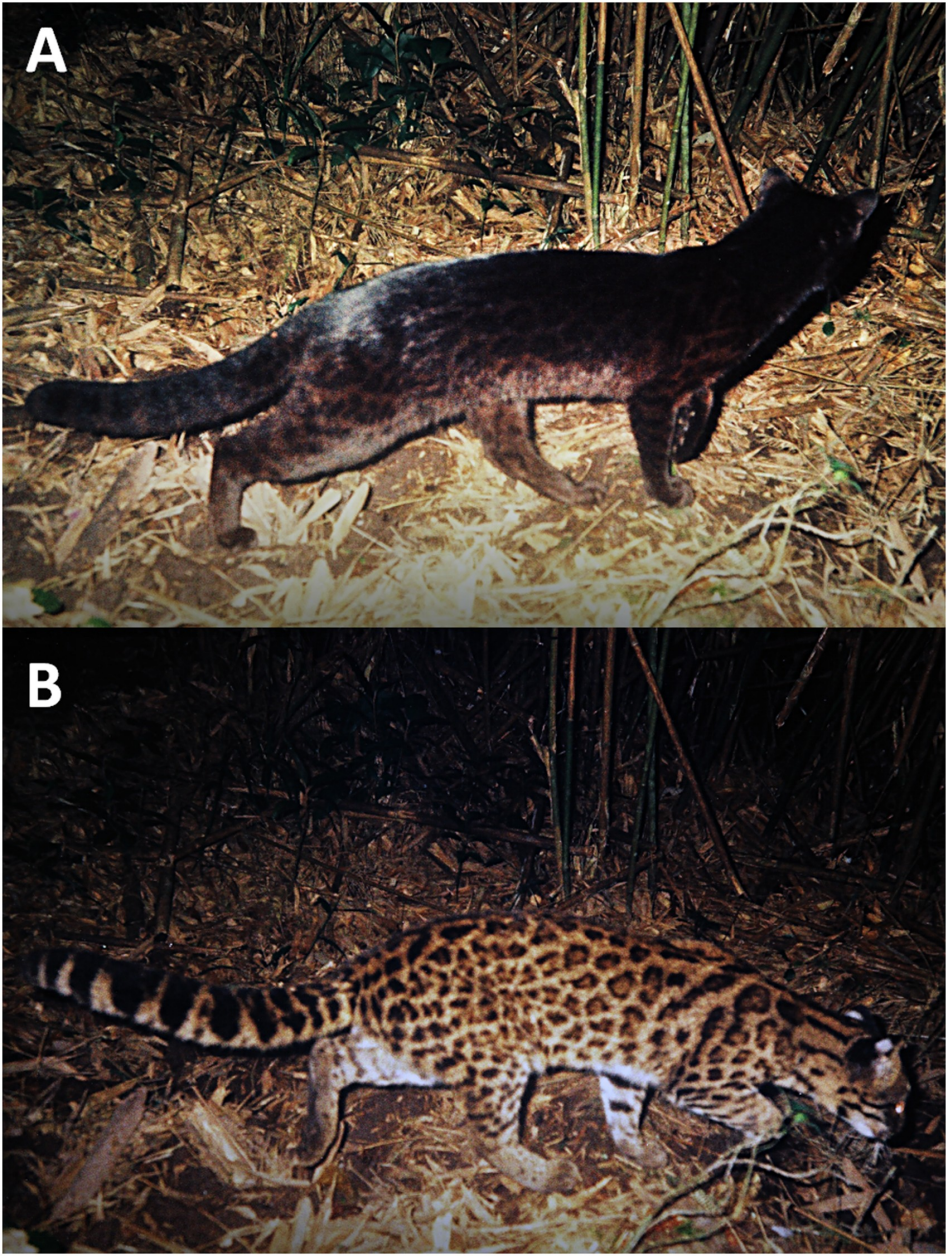
Camera-trap photographs of Southern tigrinas. (A) A melanistic individual without white ear marks; (B) A non-melanistic individual showing the white marks on the posterior surface of the ears.

Communication between conspecifics through body marks is considered a relevant factor driving phenotypic evolution [13, 14], implying that visual communication is impaired when marks on the body are absent, such as in melanistic individuals (Fig 1A). Such type of communication is especially important under conditions of low lighting, as observed in felids [15] and owls [16, 17]. Since all cat species have at least partial nocturnal activity, and most live in closed environments [6], investigation of visual communication between conspecific melanistic and/or non-melanistic individuals becomes important for understanding felid behavior and evolution. White color is conspicuous at night and in closed environments [15, 16] and, when it occurs on appendages, such as behind ears and at tail tips, it is thought to be associated with intraspecific communication (Fig 1B; S1 Video) related to threat displays, alarm postures, and/or localization/orientation behaviors [15].

White markings associated with intraspecific communication may help explain the evolution of divergent coat patterns related to visual communication and circadian cycle combined. Unlike acoustic and chemical signals, which may reach a potential prey or a predator, even if its view is obstructed [18, 19, 20], visual signals decrease the possibility of prey/predator intercepting a message intended for closer conspecifics warning about their presence. In turn, acoustic and chemical communication play important roles in advertising territory occupation and ownership [18, 20, 21]. This highlights white marks, as well as acoustic and chemical communication, as functional traits that may increase the fitness of organisms and/or their influence on other organisms and on ecosystem functions [22]. In this context, here we investigated possible associations between body markings and eco-behavioral traits to assess the adaptive implications of melanism for inter-individual communication.

Considering that melanistic individuals could be more effectively cryptic at night than non-melanistic individuals (conferring better camouflage against visual predators and ambush prey than their competitors’ [23]), and that non-melanistic individuals can communicate visually via white marks (conferring greater survival to offspring of non-melanistic mothers), this combination can help explain the evolutionary persistence of polymorphisms due to balancing selection, when both morphs gain benefits.

Four hypotheses of association of body markings with communication in felids were assessed: (1) white markings are associated with communication in felids living in closed environments [15, 24]; (2) melanistic phenotypes limit communication under low visibility at night due to the absence of white markings; (3) melanistic individuals depend on individuals with white marks to communicate; (4) communication by white markings (mediated by other individuals) facilitates the persistence of melanistic animals in closed environments. As a consequence of our hypotheses, we expect that the analysis of evolutionary models will support the coordinated evolution of traits (white markings on the ears, arrhythmic activity during diurnal and nocturnal periods, and occurrence in closed environments) in species with melanism, contributing to the knowledge of functional traits (i.e. addressing the Raunkiaeran gap) and their evolutionary history (addressing the Darwinian gap) [25].

## Materials and methods

We used data of confirmed occurrences of melanism in each Felidae species [26], avoiding anecdotal information, and considered the record of melanism in the Northern tigrina, based on the holotype of *Felis carrikeri* (Allen 1904) ([27]: p.359), and the record of melanism in the African golden cat, based on several recent camera trap records (S1 and S2 Files).

**Fig 2:**
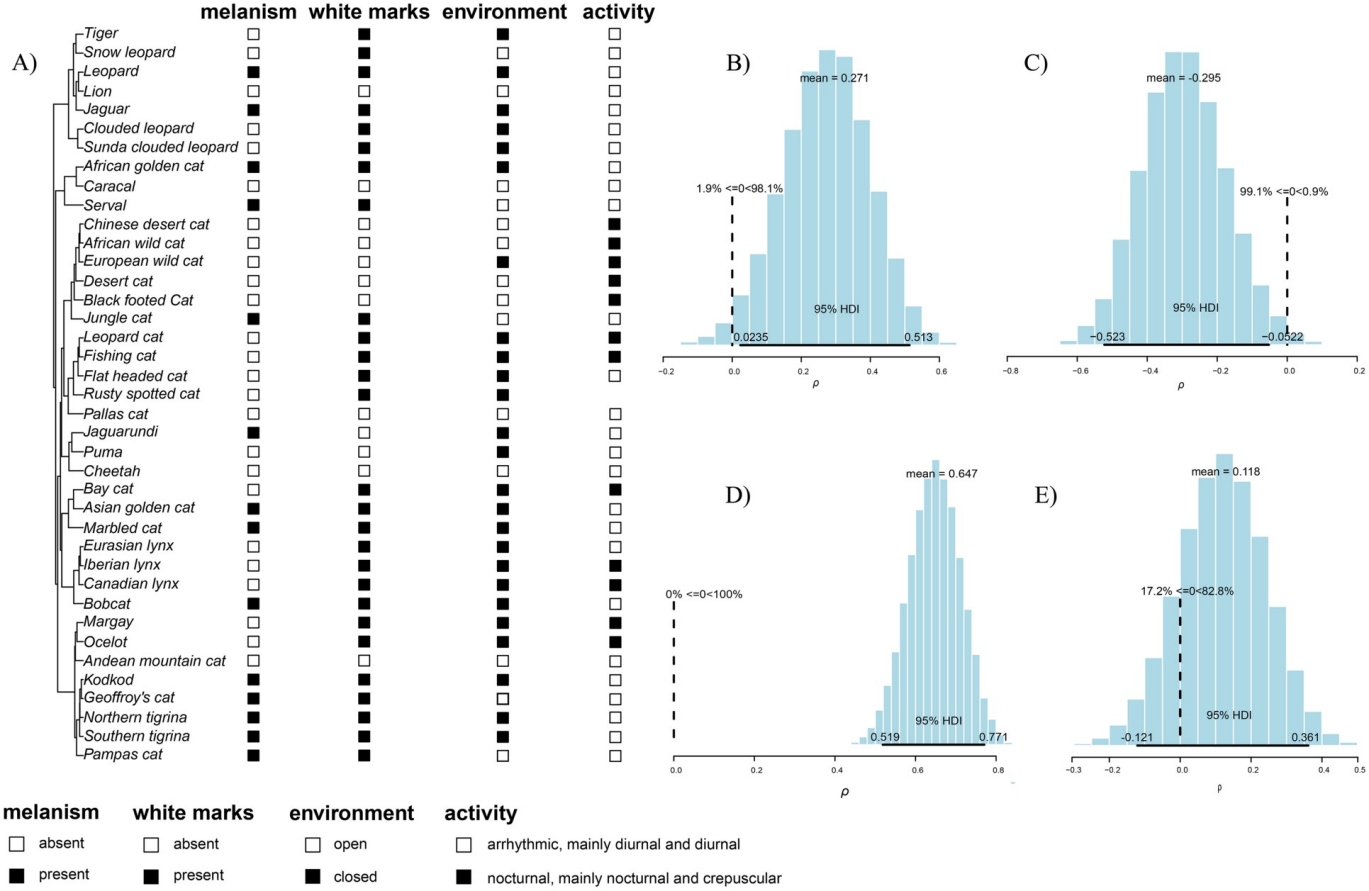
**A) Distribution of melanism, white marks and ecological variables across the phylogeny of the Felidae. B) Posterior distribution of r-values for the relationship between the presence of white marks on the ears (black quadrat) and preference for closed environments (black quadrat). C) Posterior distribution of r-values for the relationship between the presence of melanism (black quadrat) and arrhythmic activity (white quadrat). D) Posterior distribution of r-values for the relationship between the presence of melanism (black quadrat) and white marks on the ears (black quadrat). E) Posterior distribution of r-values for the relationship between the presence of melanism (black quadrat) and closed environments (black quadrat)**. HDI = Highest Density Intervals.

Therefore, melanism was documented in 15 of the 40 Felidae species (Fig 2A; S1 and S2 Files), including the domestic cat. We considered the species with white marks on the backs of ears based in Galván [24] and included African wild cat and Southern tigrina in our analysis (Fig 2A; S1 and S2 Files). We considered the record of white marks on the backs of ears in the Pampas cat, based on available images on the internet (S2 File). We searched books, articles and the internet (S2 File) for available images of potentially relevant signals provided by white marks on the backs of ears in these three species. The search on the internet was conducted mainly using Arkive (www.arkive.com) or Google Images using scientific and common names as search terms, with images being considered only when the identification at species level was validated (S2 File). We considered 27 species with white marks on the backs of ears (Fig 2A; S1 File). Melanistic individuals do not have marks or they are very weak, except for only one population of Asian golden cat, in which individual have white markings under the tail ([15, 28]; see Discussion). The domestic cat was removed from all analyses, since it may not be subjected to the same patterns of natural selection as the remaining species (S1 File).

Ecological data were obtained from Table S2 in Allen et al. [6] and Figure 2 in Galván [24], except when indicated (S2 File). Reliable information on circadian activity was not found for the rusty-spotted cat and therefore this species was not considered in the analyses that required this information (Fig 2A; S1 and S2 Files). Both response and predictors were coded as binary variables to meet the requirements of our analytical approach. Specifically, the ecological variable ‘circadian activity’ was coded as arrhythmic/nocturnal, and ‘environmental preference’ as open/closed ([6, 24], and studies cited in S2 File), as follows. A species was considered nocturnal (coded as 1) when more than 90% of the circadian activity was categorized as crepuscular and/or nocturnal; otherwise, the species were considered arrhythmic and coded as 0 (S1 and S2 Files). Environmental preference associated to open environments was coded as 0; the remaining environments were considered closed and coded as 1 (S1 File). Species found on both closed and open environments were coded as 1 [24]. Closed habitats comprised forests, rain forests, riparian or brush and scrub habitats, while open habitats comprised grasslands, deserts, and arctic habitats [15, 29].

We explored the evolutionary correlation between melanism, white markings and ecological variables using a quantitative genetic threshold model [30, 31], implemented in the phytools package (*threshBayes* function; [32]). Evolving by Brownian motion, the model assumes that, for each discrete variable there is an unobserved quantitative character (i.e. liability) [33]; so, when liability exceeds a fixed threshold, the character changes state (e.g. absence of melanism to presence of melanism). Thus, this model allows one to estimate the evolutionary correlation between variables that co-vary based on an unobserved underlying liability [31, 33].

For each hypothesis, we ran a separate Markov Chain Monte Carlo (MCMC) chain spanning 8 x 10^6^ to 1 x 10^7^ generations (sampling every 300 to 500 steps), with 2 x 10^6^ to 4 x 10^6^ generations discarded as burn-in, depending on the tested hypothesis. Qualitative (e.g. trace plots) and quantitative [34, 35] diagnostics were used to evaluate the convergence of chains and autocorrelation between adjacent samples. We reported the mean correlation from the posterior probability distribution and used the quantile function in R to obtain 95% confidence intervals. We considered that a relationship was significant when the estimated correlation coefficients differed significantly from zero (i.e. the 95% Highest Density Intervals [HDI] did not include zero). This set of analyses was conducted in the R environment [36], using functions from packages Phytools [37], APE [38], Phylobase [39] and CODA [40].

To further test whether melanism could compromise felid communication under specific conditions, we contrasted distinct models of either independent or coordinated/dependent character evolution [41, 42], with methods currently available for up to three binary traits. In such models, we contrasted whether trait states evolve independently (i.e. melanism evolving independently from the presence of body marks, or the species’ environment or its circadian behavior) or in a coordinated way (i.e. melanism evolving only if the species has a particular combination of body marks, environment, and circadian behavior). We carried out two sets of analyses to assess whether evolution was independent or coordinated for either 1) melanism, body marks, and the type of circadian behavior or 2) melanism, body marks, and the type of environment. Persistence time in each state was used to indicate suitability of trait combinations, where persistence was defined as the inverse of the sum of the transition rates away from a given character state. The characterization of types of evolutionary models is available in S3 File.

Model selection was based on the small-sample-size corrected version of the Akaike information criterion (AICc) and AIC weights (*w*_*i*_). Transition rates between two binary traits of all model parameters were estimated by maximum likelihood using the corHMM package in R. In both analyses, we used a dated, ultrametric phylogeny [43].

## Results

**Fig 3:**
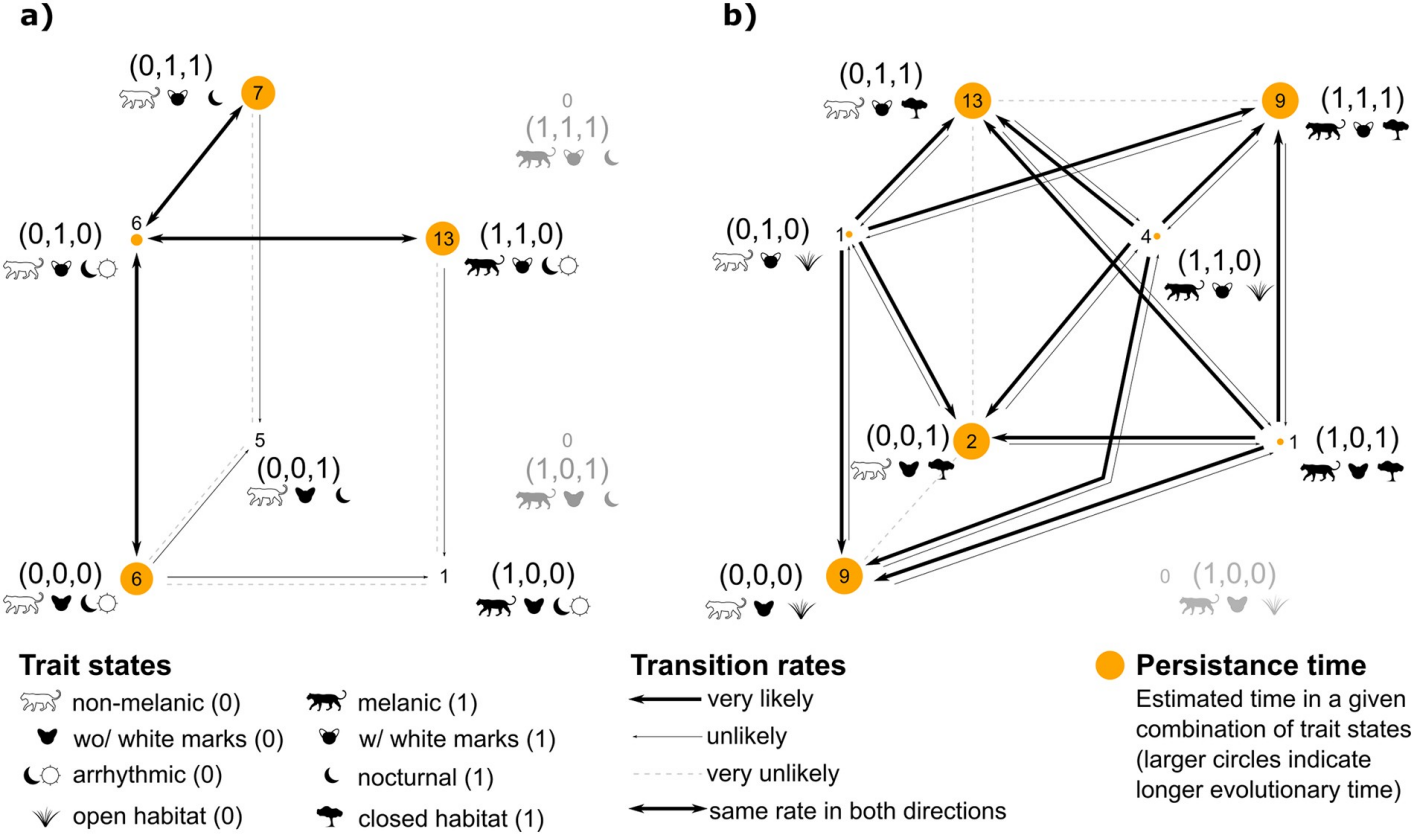
Most likely evolutionary pathways and transition rates between combinations of species traits and melanism for 39 cat species. A) The independent evolution of white marks on the back of ears (or their absence), circadian activity (arrhythmic or nocturnal) and melanism. B) The independent evolution of white marks on the back of ears (or their absence), environment preference (open or closed habitat) and melanism. Solid lines and arrows are proportional to the transition rates between possible states (wider lines indicate higher rates). Dashed lines indicate nearly null rates and missing lines indicate that the transition was not tested because of unobserved trait combinations. The size of each circle is proportional to the persistence time in that state, where persistence time is defined as the inverse of the sum of the transition rates away from a given character state (see Figure 2 in [45]).

The 14 wild species of Felidae (~36%) showing melanism (excluding the domestic cat) were arrhythmic regarding the circadian cycle. Of those, 10 species (~71%) were associated with closed environments, and 13 species (~93%) exhibit white marks on the backs of ears (Figs 2A, 3A and 3B; S1 and S2 Files). Although occurring in seven of the eight felid lineages (the exception being the Leopard cat lineage *sensu* Li et al. [43]), and not considering the molecular evidence for multiple origins of this trait in the Felidae [3, 26, 44], melanism was inferred from this data set to be as likely as the ‘normal’ (non-melanistic) phenotype as the ancestral state in felids (when considered apart from other traits in the ancestral character reconstruction analysis—ACR, S1 Fig).

With respect to ecological features, the arrhythmic activity can be considered as a more plausible condition for the ancestor of *Panthera*, *Caracal*, *Puma*, Bay cat and Ocelot lineages, and the nocturnal activity for the ancestor of Domestic cat, Leopard cat and *Lynx* lineages (ACR, S2 Fig). Occurrence in closed *versus* open environments is equally balanced across the entire felid phylogeny (ACR, S3 Fig). The white marks on the backs of ears were probably the ancestral condition in felids, becoming absent in Domestic cat and *Puma* lineages (ACR, S4 Fig).

### Testing the hypothesis of communication by body markings

After taking phylogenetic relationships into account, white marks were associated with species living in closed environments (r-value mean = 0.271 [HDI = 0.023–0.513]) (Fig 2B). Felidae species exhibiting melanism were associated with arrhythmic activity (r-value mean = -0.295 [HDI = -0.052 –-0.523]) (Fig 2C) and melanism was associated with species in which the “normal phenotype” has white marks (r-value mean = 0.647 [HDI = 0.519–0.771]) (Fig 2D). Regarding habitat preference, species with melanism were not correlated with closed environments (r-value mean = 0.118 [HDI = -0.121–0.361]) (Fig 2E).

### Evolutionary models for melanism and associated traits

Comparative analysis of several evolutionary models indicated more support for the evolution of melanism to be coordinated with the evolution of circadian habits and white marks (ΔAICc < 2) (Fig 3A; S1 Table). The best model also indicated higher support for different transition rates for suitable (melanism, white marks, and arrhythmic habits) and unsuitable traits (melanism without white marks), especially after removing highly unfavorable trait combinations (melanism and nocturnal habits) from the analyses (ΔAICc = 0; *w*_i_ = 0.559). There were three alternative groups of trait states in the model (Fig 3A; S1 Table; S3 File). The first was composed by six species without melanism (with white marks and arrhythmic) with low persistence time, and high transitions rates towards to second group with high persistence time and high transitions rates, that include most species with melanism (with white marks and arrhythmic) and species without melanism, with or without white marks, and nocturnal or arrhythmic. The third group, with low to absent transitions between groups and low persistence time, is composed of the *Felis* lineage (without melanism, without white marks, and nocturnal) and jaguarundi (with melanism, without white marks, and arrhythmic) (Fig 3A). The evolution of melanism being independent of the evolution of circadian habits and white marks had secondary support in the analysis (ΔAICc = 1.1; *w*_*i*_ = 0.319) (Fig 3A; S1 Table).

Considering the potential association of the evolution of melanism and environment covarying with communication by white marks, the best model indicates more support for the independent evolution of traits (ΔAICc < 2) (Fig 3B; S1 Table). The best model also indicated higher support for “three types” of trait combinations (suitable/intermediate/unsuitable traits), especially after removing highly unfavorable trait combinations (melanism, without white marks, and open habitat) (ΔAICc = 0; *w*_i_ = 0.422) (S1 Table; S3 File). Here two groups of trait combinations were observed in the model, but now the transition rates between the alternative groups were different. The first group, including five species with melanism (with white marks and open habitat: jungle cat, Geoffroy’s cat, Pampas cat, and serval; and without white marks, and closed habitat: jaguarundi) present low persistence time and high transition rates toward the alternative group; the second group, with the remaining species, including most species with melanism (with melanism, with white marks, and closed habitats), presents high persistence time and low transition rates towards the first alternative group (Fig 3B]. The evolution of melanism being coordinated with the evolution of environmental preference and white marks had secondary support in the analysis (ΔAICc = 1.2; *w*_*i*_ = 0.227) (Fig 3B; S1 Table).

## Discussion

Melanism is a widespread phenomenon in the animal kingdom and has long been used to investigate evolutionary change (e.g. [2, 12]). The occurrence of melanism is common in the Felidae [46], having been documented in 14 out of its 40 wild species ([3, 26, 27], this study), and being absent in only one out of eight major lineages (the Leopard cat lineage) [S1 and S2 Files].

Research on melanism in felids has so far focused mostly on its genetics [3, 26, 44] and ecology [4, 5, 6, 46, 47], but so far had not addressed the role of visual communication in the evolution of this phenotype. The results presented here support our first three hypotheses: (1) Behavior plays an important role in the occurrence of melanism, since white marks on the back of the ears are associated with communication in felids living in closed environment ([15, 24], this study) (Fig 2B). We infer that this relationship derives from the fact that while marks allow communication at short distances and cannot be perceived at greater distances by potential prey or predators, as could occur in open environments. (2) Melanistic phenotypes limit the communication under low visibility at night due to the absence of white markings, thus being associated with species that do not present exclusively nocturnal activity (Fig 2C). We infer that this pattern occurs because the survival of cubs/kittens with a melanistic mother would be hampered by limited visual communication between mother and cubs/kittens under low-light conditions. (3) Melanistic individuals depend on individuals with white marks (“normal” phenotype) to communicate (Fig 2D), since melanistic cubs/kittens depend on their mothers with “normal phenotypes” and white marks.

Nevertheless, the results did not support our fourth hypothesis, that melanism is associated with closed habitats (Fig 2E), allowing melanistic individuals to use more closed environments (where melanism is cryptic) than open environments (where melanism is conspicuous). Unlike previous studies focusing on ecological analyses of individual species [4, 46, 47], our approach did not recapitulate this pattern, increasing the evidence that a broader evolutionary perspective tends not to detect this association [5, 6], likely due to other factors strongly affecting the long-term dynamics of this phenotype. The Felidae includes some of the most elusive species among mammals [6], with all species presenting some nocturnal habits. Therefore, crypsis could not only be a selective advantage related to spatial niches, such as the association of melanistic jaguarundis and moist forests [46], but can also be associated with the temporal niche, since melanistic individuals could be more effectively cryptic on bright nights than the “normal” phenotype [23].

Therefore, if camouflage may be considered an advantage of melanistic individuals, the absence of communication by white marks can be considered their Achilles’ heel, resulting in an evolutionary dilemma for these cryptic individuals, i.e. to be almost invisible at night, but not to communicate visually in dim light. In the long run, both morphs gain benefits, helping explain the evolutionary persistence of such a polymorphism due to balancing selection.

The evolutionary analysis provided more support to the coordinated rather than independent evolution of melanism regarding circadian behavior and white marks (Fig 3A; S1 Table), suggesting that changes in a trait are related to the other traits considered at the same time. Such correlation fits the pattern of inferred relationships inferred in our other analyses. Although melanism evolved several times independently in the cat family [3, 26, 44] and was positively selected in at least some cases, it would be expected that melanistic individuals within species not presenting white marks and arrhythmic activity would have very low fitness, making melanism disappear quickly from such populations. Over time, such fitness differences would lead to the recurrent combinations observed in the Felidae. In contrast, there was more support to the independent rather than coordinated evolution of melanism with respect to the preferential environment and white marks (Fig 3B; S1 Table), possibly due to the absence of significant relationship between melanism and closed habitats (Fig 2E).

The ancestral conditions of melanism and circadian activity were inconclusive in our analyses. Still, it may be assumed that nocturnal habits were an ancestral condition [48] and melanism is a derived condition in felids [3, 26, 46]. Considering the relationship between melanism and more diurnal habits (6, this study), it is plausible to suppose that conditions for the appearance of melanism are currently more common, since mammals have become more diurnal throughout evolution [48], and most felids have habits that are mainly arrhythmic, independent of the presence of white markings.

The jaguarundi may be considered one exception that corroborates our hypothesis, due to the absence of white marks. Being the most diurnal species among the felids [49], the nocturnal communication by white marks does not seem to be vital to its survival, and thus melanism can reach high frequencies (≈ 80%) in populations found in moist forests [46], suggesting that this species would have a trend to persist with the current combination (melanism, absence of white marks, arrhythmic activity and closed habitat)(Fig 3A and 3B). The need for white markings as a method of visual communication in low visibility conditions may limit the melanistic phenotype in most felid species (see [1, 3]). However, if species or populations are diurnal, such as jaguarundi [49] and leopards [50, 51, 52], respectively, then melanism may persist with limited negative consequences on communication.

In leopards the frequency of melanistic individuals varies significantly among populations. A very low incidence of melanism has been observed in the open savannas of Africa (1.25%) [1], but reaches 50% of frequency to near fixation in moist forests of the Malay Peninsula [1, 4, 52]. Interestingly, while populations found in the Malay Peninsula Forests are mainly diurnal [51], African populations display diurnal and nocturnal activity [53].

Such change in the circadian behavior of leopards seems to be linked to the higher abundance of tigers (which are nocturnal) in the Malay Peninsula, resulting in leopard activity changing to diurnal in that region [51]. Javan leopards are also believed to be mainly black [54] and diurnal [50]. These conditions seem to be a way to avoid conflicts with larger felid species [23, 55], and may, in turn, have influenced the frequency of melanism in those regions. Like these leopards, one population of Asian golden cats in Sikkim, India, also corroborated our hypotheses, since all individuals presented melanism and white marks under the tail [28], a condition also associated with intraspecific communication [15], an exception that, according to our hypotheses, would allow visual communication between individuals under low lighting conditions and, eventually, the fixation of these characteristics.

Our results corroborate the hypothesis that the absence of white markings on the backs of ears in the melanistic phenotype is a limiting condition for communication under low visibility, supporting the coordinated evolution of melanism with these white marks and also with arrhythmic activity. Scientists have long believed that crepuscular and nocturnal animals forgo visual signals and rely solely on sound and chemical communication, but many cases seem to indicate otherwise [19]. White marks of feathers and fur on a dark background have the potential to be used to communicate with conspecifics in dim light at night ([19]; this study) in closed habitats ([14, 24]; this study), influencing important population processes and entailing selective advantages that may promote the ecological success of polymorphic species [56].

However, if natural selection provided adaptive advantages to some polymorphisms [56], anthropogenic changes to the environment may contribute to change the circadian activity patterns of the populations [57], in turn affecting the frequency of melanistic and non-melanistic individuals. This might lead to the disappearance of the “normal” phenotype, similar to the case of leopards in Southeast Asia [52], or the disappearance of melanistic individuals, as in the case of Southern tigrina in southern Brazil ([23, 55], Unpublished data). Therefore, studies of circadian activity that include changes in prey availability and the in the abundance of other carnivore species in a community may contribute to better understand the observed variation in melanism frequencies in felid populations, and ultimately to the conservation of these threatened taxa [58].

This study showed for the first time the importance of visual communication under conditions of low lighting for the adaptive dynamics of melanism in felids, suggesting that white markings behind the ears have contributed to the evolutionary success of felids as nocturnal predators in forested environments. By highlighting the role of this additional factor affecting the dynamics of melanism, it opens up a new path for research on the ecology and evolution of mammalian polymorphic coloration.

## Supporting information

S1 File Database of morphological (melanism and body marks) and ecological (circadian activity and environmental preference) features of felids. (XLSX)

S2 File Database references obtained from multiple search engines describing felid characteristics. (XLSX)

S3 File Characterization of types of evolutionary models. (PDF)

S1 Fig Phylogenetic analysis of melanism in the cat family. (PDF)

S2 Fig Phylogenetic analysis of circadian activity in the cat family. (PDF)

S3 Fig Phylogenetic analysis of environment preference in the cat family. (PDF)

S4 Fig Phylogenetic analysis of body marking in the cat family. (PDF)

S1 Table Model selection for the relationship between melanism and communication by body marks, circadian behavior, and environment type. (PDF)

S1 Video Typical alert display showing the white marks on the posterior surface of the ears in Southern tigrina. (MP4)
