# Supplementary material for: Melanism evolution in the cat family is influenced by intraspecific communication under low visibility
Source: PLoS One. 2019 Dec 18;14(12):e0226136. doi: 10.1371/journal.pone.0226136 (PMC6919575; doi:10.1371/journal.pone.0226136)
Supplement: S3 File — (PDF) [file pone.0226136.s003.pdf]

Characterization of types of evolutionary models

For each set of three traits and in addition to setting either independent or coordinated evolution, we contrasted three types of evolutionary models: 1) distinct rates of evolution between states and no restriction in trait state combinations; 2) distinct rates of evolution and restrictions in trait states by removing very unfavorable combinations (see below); and 3) a single rate of evolution between states and restriction in character state by removing highly unfavorable combinations. We considered as very unfavorable combinations those that could represent key selective disadvantages. Persistence time in each state was used to indicate suitability of trait combinations, where persistence was defined as the inverse of the sum of the transition rates away from a given character state. Nocturnal species being melanistic (disregarding whether they show or not body markings) and the presence of white marks when living in an open environment were here considered as very unfavorable combinations states. Because of convergence problems with full parameterized models, we shrunk parameters by separating phenotypes as either suitable/unsuitable ("two types" of trait combinations) or suitable/intermediate/unsuitable ("three types" of trait combinations). Unsuitable states consisted of highly unfavorable trait combinations already mentioned and, for example, we considered a selective disadvantage to show conspicuous white marks in open environments [see Ortolani et , 1999]) (disregarding melanism). Each model of coordinated evolution was also contrasted to models of independent evolution of traits.

Suitability of morphological and ecological characteristics for melanism in Felid species (Melanism, White marks, and Circadian Activity).

Melanism (Melanism= 1; No Melanism = 0)	White ear/tail (White = 1; No White = 0)	Activity_time (Arritimico = 0; Noturno=1)	Suitability	Legend for Suitability
0	0	0	2	1-unsuitable
1	0	0	1	2-intermediary
0	1	0	2	3-suitable
0	0	1	1	
1	1	0	3	
1	0	1	1	
0	1	1	2	
1	1	1	1	

Suitability of morphological and ecological characteristics for melanism in Felid species (Melanism, White marks, and Environmental Preference).

Melanism (Melanism= 1; No Melanism = 0)	White ear/tail (White = 1; No White = 0)	Environment (Closed=1; Open=0)	Suitability	Legend for Suitability
0	0	0	2	1-unsuitable
1	0	0	1	2-intermediary
0	1	0	1	3-suitable
0	0	1	1	
1	1	0	1	
1	0	1	1	
0	1	1	2	
1	1	1	3	
