# Supplementary figures and images for: Melanism evolution in the cat family is influenced by intraspecific communication under low visibility
Source: PLoS One. 2019 Dec 18;14(12):e0226136. doi: 10.1371/journal.pone.0226136 (PMC6919575; doi:10.1371/journal.pone.0226136)

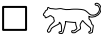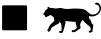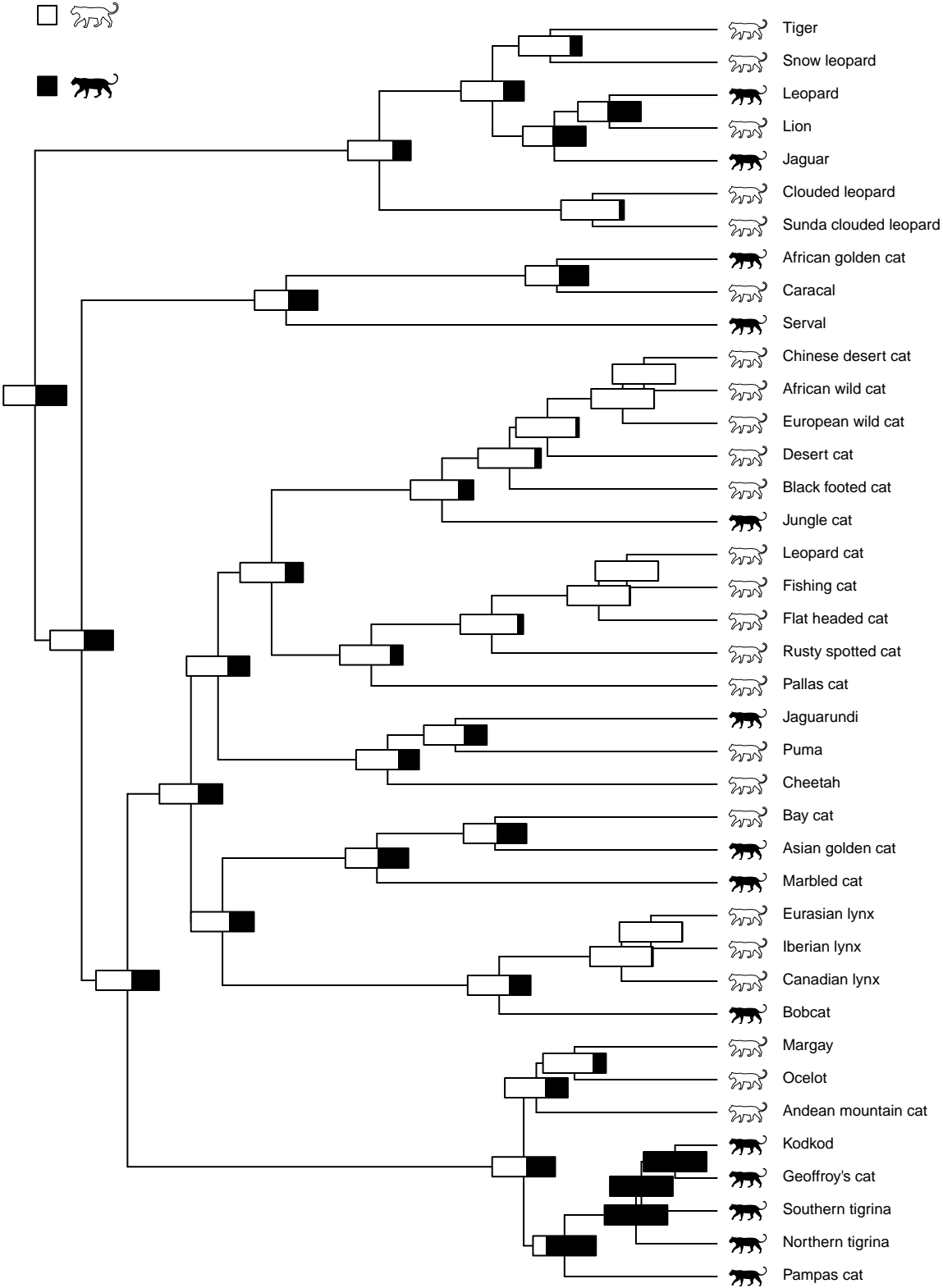

Supplement: S1 Fig — (PDF) [file pone.0226136.s004.pdf]

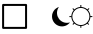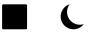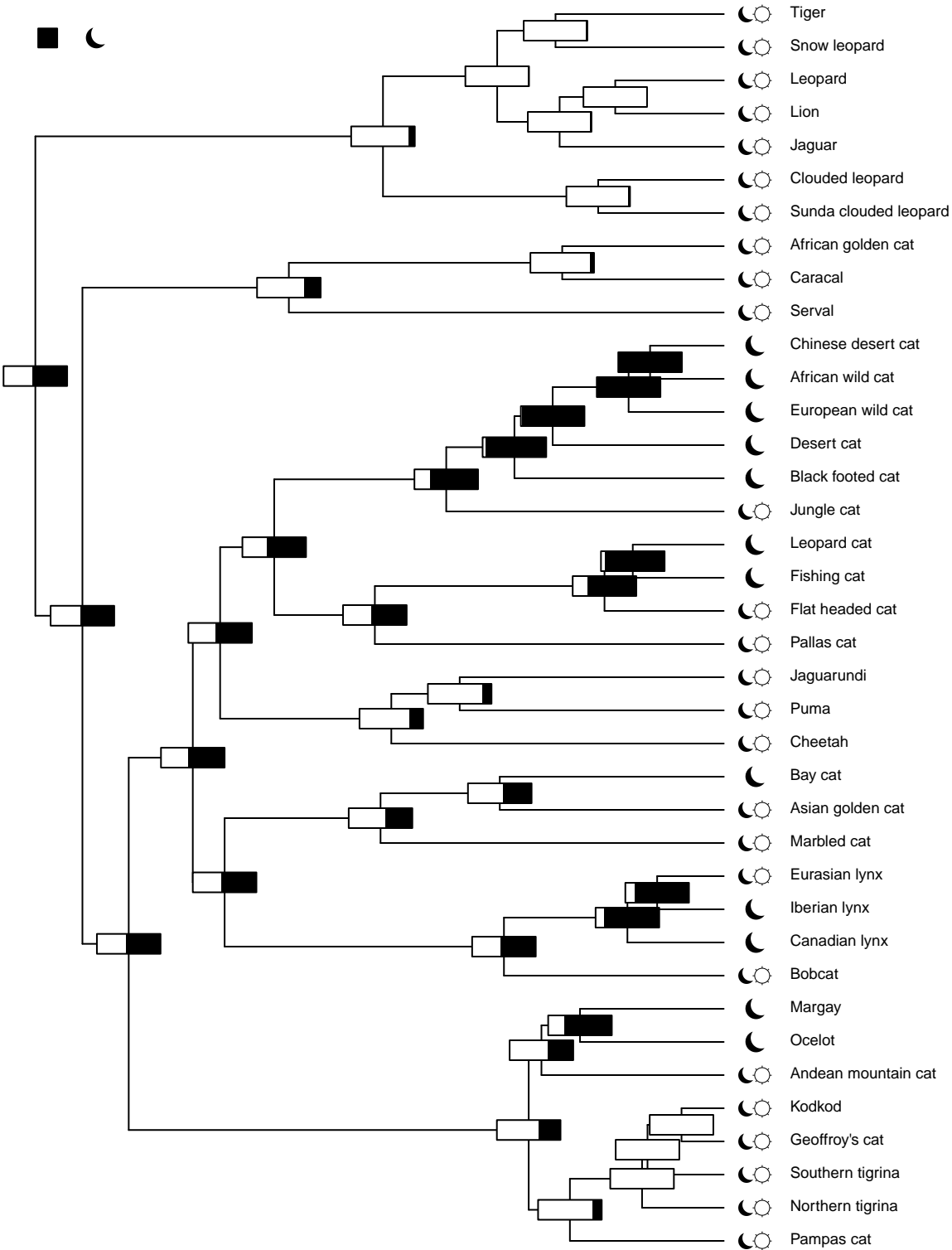

Supplement: S2 Fig — (PDF) [file pone.0226136.s005.pdf]

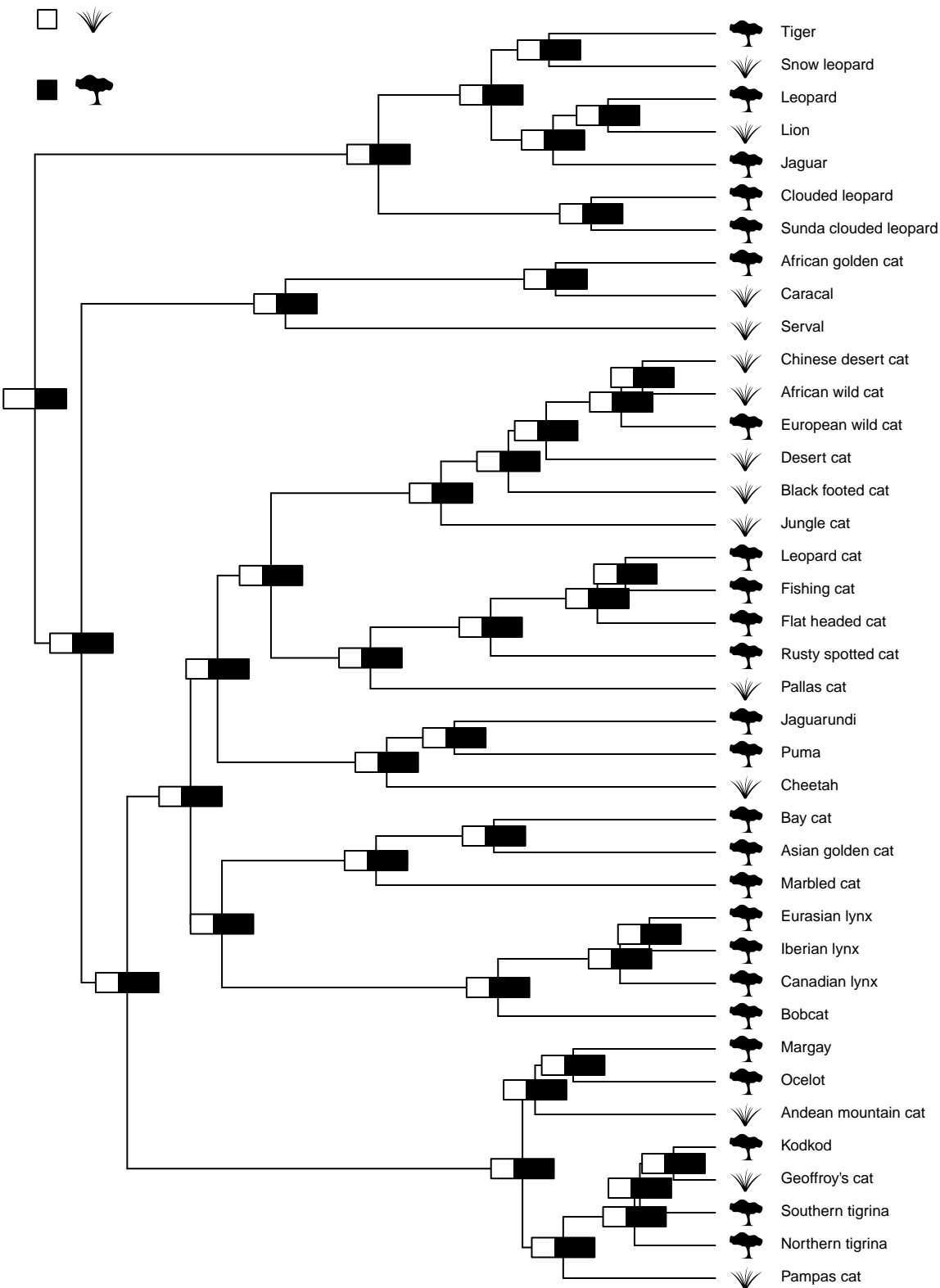

Supplement: S3 Fig — (PDF) [file pone.0226136.s006.pdf]

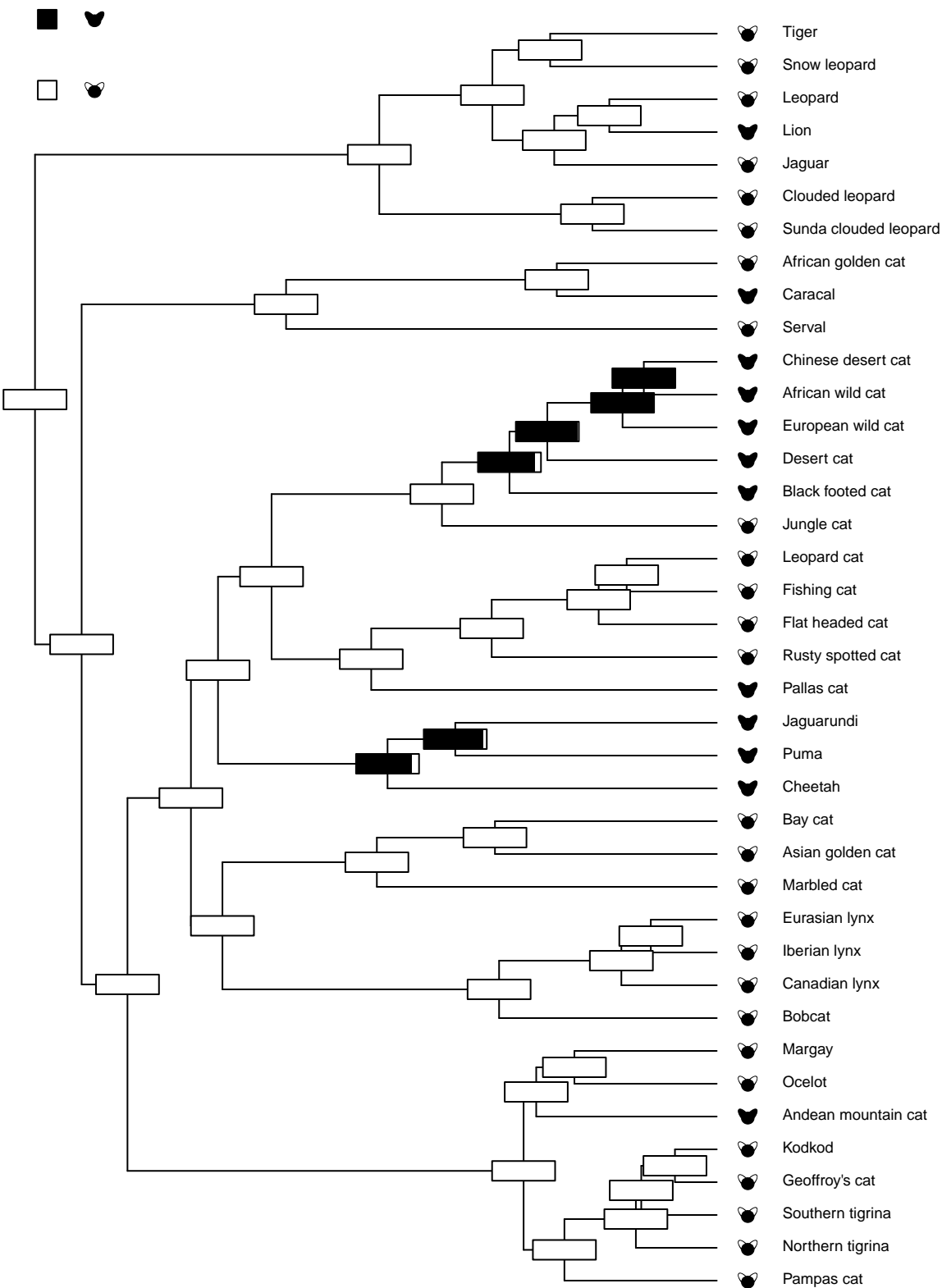

Supplement: S4 Fig — (PDF) [file pone.0226136.s007.pdf]
