# Supplementary material for: Melanism evolution in the cat family is influenced by intraspecific communication under low visibility
Source: PLoS One. 2019 Dec 18;14(12):e0226136. doi: 10.1371/journal.pone.0226136 (PMC6919575; doi:10.1371/journal.pone.0226136)
Supplement: S1 Table — (PDF) [file pone.0226136.s008.pdf]

Melanism, body marks, and type of circadian behavior

Evolutionary model	Phenotypes	Restrictions	Parameters	–ln L	AIC	ΔAIC	w_i
Coordinated	Two types	Unobserved removed	3	-52.838	112.3	0.0	0.559
Independent	Two types	Unobserved removed	3	-53.397	113.5	1.1	0.319
Independent	Three types	Unobserved removed	3	-54.973	116.6	4.3	0.066
Coordinated	Three types	Unobserved removed	3	-56.160	119.0	6.6	0.020
Independent	Two types	None	3	-56.495	119.7	7.3	0.014
Independent	All equal	Unobserved removed	1	-58.037	120.4	8.1	0.010
Coordinated	Two types	None	3	-57.274	121.2	8.9	0.007
Independent	Three types	None	3	-57.985	122.6	10.3	0.003
Coordinated	All equal	Unobserved removed	1	-60.552	125.4	13.1	0.001
Coordinated	Three types	None	3	-60.038	126.7	14.4	0.000
Independent	All equal	None	1	-64.517	133.4	21.0	0.000
Coordinated	All equal	None	1	-66.053	136.4	24.1	0.000

Melanism, body marks, and environment type

Evolutionary model	Phenotypes	Restrictions	Parameters	–ln L	AIC	ΔAIC	w_i
Independent	Three types	Unobserved removed	3	-62.547	131.8	0.0	0.422
Coordinated	Three types	None	3	-63.164	133.0	1.2	0.227
Coordinated	Three types	Unobserved removed	3	-63.615	133.9	2.1	0.145

Independent	Three types	None	3	-63.653	134.0	2.2	0.139
Independent	All equal	Unobserved removed	1	-66.586	137.5	5.7	0.024
Independent	Two types	Unobserved removed	1	-66.586	137.5	5.7	0.024
Independent	Two types	None	3	-66.602	139.9	8.1	0.007
Coordinated	Two types	None	3	-66.844	140.4	8.6	0.006
Coordinated	All equal	Unobserved removed	1	-68.942	142.2	10.4	0.002
Coordinated	Two types	Unobserved removed	1	-68.942	142.2	10.4	0.002
Independent	All equal	None	1	-70.125	144.6	12.8	0.001
Coordinated	All equal	None	1	-70.940	146.2	14.4	0.000
